# Methanolic Extract of *Rhizoma Coptidis* Inhibits the Early Viral Entry Steps of Hepatitis C Virus Infection

**DOI:** 10.3390/v10120669

**Published:** 2018-11-27

**Authors:** Ting-Chun Hung, Alagie Jassey, Chien-Ju Lin, Ching-Hsuan Liu, Chun-Ching Lin, Ming-Hong Yen, Liang-Tzung Lin

**Affiliations:** 1 Graduate Institute of Natural Products, College of Pharmacy, Kaohsiung Medical University, 807 Kaohsiung, Taiwan; h5911121@ms33.hinet.net (T.-C.H.); cclin.herba@gmail.com (C.-C.L.); 2 Department of Clinical Pathology, Chi Mei Medical Center, Tainan 710, Taiwan; 3 International Ph.D. Program in Medicine, College of Medicine, Taipei Medical University, Taipei 110, Taiwan; alagie_jassey@yahoo.com; 4 School of Pharmacy, College of Pharmacy, Kaohsiung Medical University, Kaohsiung 807, Taiwan; mistylin@kmu.edu.tw; 5 Graduate Institute of Medical Sciences, College of Medicine, Taipei Medical University, Taipei 110, Taiwan; julia.chliu@gmail.com; 6 Department of Microbiology & Immunology, Dalhousie University, Halifax, NS B3H 4R2, Canada; 7 Department of Microbiology and Immunology, School of Medicine, College of Medicine, Taipei Medical University, Taipei 110, Taiwan

**Keywords:** HCV, *Rhizoma coptidis*, herbal medicine, antiviral, entry inhibition

## Abstract

Hepatitis C Virus (HCV) remains an important public health threat with approximately 170 million carriers worldwide who are at risk of developing hepatitis C-associated end-stage liver diseases. Despite improvement of HCV treatment using the novel direct-acting antivirals (DAAs) targeting viral replication, there is a lack of prophylactic measures for protection against HCV infection. Identifying novel antivirals such as those that target viral entry could help broaden the therapeutic arsenal against HCV. Herein, we investigated the anti-HCV activity of the methanolic extract from *Rhizoma coptidis* (RC), a widely used traditional Chinese medicine documented by the WHO and experimentally reported to possess several pharmacological functions including antiviral effects. Using the cell culture-derived HCV system, we demonstrated that RC dose-dependently inhibited HCV infection of Huh-7.5 cells at non-cytotoxic concentrations. In particular, RC blocked HCV attachment and entry/fusion into the host cells without exerting any significant effect on the cell-free viral particles or modulating key host cell entry factors to HCV. Moreover, RC robustly suppressed HCV pseudoparticles infection of Huh-7.5 cells and impeded infection by several HCV genotypes. Collectively, our results identified RC as a potent antagonist to HCV entry with potential pan-genotypic properties, which deserves further evaluation for use as an anti-HCV agent.

## 1. Introduction

Hepatitis C virus (HCV) is an important liver pathogen belonging to the *Flaviviridae* family with an enveloped positive single-stranded RNA genome. HCV has seven genotypes (genotype 1~7) and a genome size of about 9.6 kb which encodes a polyprotein that is approximately 3000 amino acids long. The polyprotein upon translation is processed by viral and host proteases to yield 10 matured protein including structural proteins, Core, E1, E2, and p7 ion channel, as well as non-structural proteins NS2, NS3, NS4A, NS4B, NS5A, and NS5B [1]. HCV entry into the host hepatocytes is mediated by interaction with several notable cell surface and tight junction receptors/co-receptors including heparin sulfate proteoglycans (HSPG), cluster of differentiation 81 (CD81), low density lipoprotein receptor (LDLR), scavenger receptor class B type I (SR-BI), claudin-1 (CLDN1), and occludin (OCLN) [2,3]. Additional factors that can influence viral entry include apolipoprotein E (ApoE), which is incorporated on infectious HCV virions [4], and can function as an exchangeable apolipoprotein between secreted ApoE-associated lipoproteins and the HCV lipoviroparticle (LVP) to enhanced HCV infection [5].

There are over 170 million HCV carriers worldwide. HCV infection can lead to chronic hepatitis, cirrhosis, and liver cancer, and there is still no effective vaccine against the virus. While the previous standard of care consisting of PEGylated-interferon (IFN)-α in combination with ribavirin is associated with several important drawbacks including severe side effects and low efficacy against HCV genotype 1, the recent introduction of the direct-acting antivirals (DAAs) targeting the viral non-structural proteins has substantially improved the sustained virological response (SVR) in the most difficult to treat genotype 1 patients [6]. However, the DAAs also have challenges including potential toxicity, especially from drug-drug interactions (DDIs). For instance, HCV protease inhibitors are at high risk for DDIs as they are known substrates and inhibitors of cytochrome P450 (CYP) 3A4 system and can interfere with the metabolism of other drugs including immunosuppressants (e.g. cyclosporine and tacrolimus) when co-administered in liver transplant setting [7]. Other drug-drug interactions from HCV DAAs include those with acid-suppression therapies (e.g. famotidine and omeprazole) or the human immunodeficiency virus (HIV) antiretroviral agents (e.g. Rilpivirine and Efavirenz), which have been shown to decrease the effectiveness of the HCV NS5A inhibitor Ledipasvir [8] and produce adverse drug reactions with the protease inhibitor Paritaprevir [7], respectively. In addition, due to the great genetic variability of HCV, selection of resistant mutants is becoming a challenge as a greater number of people are being treated in real-world settings, which can potentially lead to DAA failures [6,9]. Therefore, continuous identification of novel candidate drugs particularly with a different mode of action to improve the current therapeutic strategies is highly envisaged.

*Rhizoma Coptidis* (RC) is the dried rhizome typically obtained from *Coptis chinensis* Franch (‘Chinese goldthread’), which is a medicinal plant of the *Ranunculaceae* family [10]. RC is one of the most commonly used Chinese medicinal herbs (also known as ‘*Huang Lian’*) documented by the WHO [10] and is known to contain various bioactive alkaloids [11]. It is traditionally used for its “heat clearing” and “detoxification” effects such as treatment against arthritis, burns, eczema, microbial infections, and gastrointestinal diseases [10,12,13]. In addition, RC’s traditional usage against infections has been correlated through several recent studies that validated its antimicrobial functions, including against several bacteria and viruses [13]. Specifically, RC and its major constituents have been shown to exert inhibitory effects against herpesvirus, respiratory syncytial virus, and mouse hepatitis A virus infections [14,15,16]. These precedents suggest that RC may be a potent source for the discovery of novel antiviral treatments. Since the effect of RC on HCV infection remains largely unexplored, and in an attempt to identify novel anti-HCV agents, in this study we examined the impact of the methanolic extract of RC on HCV infection. Our results demonstrated that RC could robustly inhibit HCV infection by targeting the early steps in viral entry. Specifically, the targeted steps included viral attachment and entry/fusion into the host cells. In addition, the RC-mediated inhibition of HCV is not genotype-specific as the drug equally inhibits other HCV genotypes, thereby identifying RC as a potential pan-genotypic anti-HCV agent.

## 2. Materials and Methods

### 2.1. Cell Culture and Virus Production

Culture of Huh-7.5 cells (human hepatoma, Huh-7 cell derivative) and production of cell-culture derived HCV particles (HCVcc) from the *Gaussia* luciferase reporter-tagged Jc1FLAG2(p7-nsGluc2A) construct (genotype 2a; kindly provided by Dr. Charles M. Rice) were carried out as previously described [17]. Virus concentration was expressed as multiplicity of infection (MOI) and the basal media for all viral infection analyses consisted of Dulbecco’s Modified Eagle’s Medium (GIBCO-Invitrogen, Carlsbad, CA, USA) containing 2% fetal bovine serum.

### 2.2. Plant Extract Preparation

*Rhizoma Coptidis* roots from *Coptis chinensis* Franch (ID#kew-2736105 from The Plant List [18]) were obtained from local pharmacy store (Kaohsiung, Taiwan) and authenticated by Dr. Ming-Hong Yen using anatomical methods as well as by HPLC analysis through comparison to known molecular standards as previously described [10,19]. A voucher specimen was deposited at the Kaohsiung Medical University herbarium (CTM-RCC03). For methanol extraction [20], the roots were washed, dried, and homogenized before extraction with 100 % methanol, followed by concentration *in vacuo*. The methanolic RC stock was dissolved in dimethyl sulfoxide (DMSO; Sigma, St. Louis, MO, USA) prior to use.

### 2.3. Cytotoxicity Assay and Antiviral Activity Analysis

Huh-7.5 cells seeded at 1 × 10^4^ cells/well in 96-well plates overnight were treated with increasing concentrations of RC for 5 days. The cells were then washed twice with phosphate buffered saline (PBS) before XTT cell viability analysis as previously described [21]. For examining antiviral activity, Huh-7.5 cells (1 × 10^4^ cells/well in 96-well plates) were concurrently treated with the virus (MOI = 0.01) and the test drug at various concentrations before incubation at 37 °C for 3 days. The anti-HCV activity was determined by measuring the luciferase reporter signals using the BioLux™ Gaussia Luciferase Assay Kit (New England Biolabs; Pickering, ON, Canada) and a luminometer (Promega; Madison, WI, USA) as previously reported [22]. Data were expressed as percent (%) HCV infectivity from test treatments relative to medium control (virus only). IFN-α (Sigma) served as positive control.

### 2.4. Time-of-Drug-Addition Assay

The time-of-drug-addition assay which provides information on the target of test agents in the viral life cycle consisted of pre-treatment, co-addition treatment, and post-infection treatment, and was performed as previously described [22]. For all analyses, Huh-7.5 cells were seeded in 96-well plates at a density of 1 × 10^4^ cells/well overnight and infection with HCVcc was carried out at MOI = 0.01. Luciferase activity for all conditions was determined as described earlier following 72 h of incubation at 37 °C.

### 2.5. Synchronized Infection Analysis on Early Viral Entry

The synchronized infection analysis to determine the effect of test agents on early viral entry was performed as previously described [22]. To examine viral inactivation, the test drug was incubated with the cell-free virus particles prior to diluting the virus-drug mixture to a subtherapeutic concentration and infecting the host cells (final HCVcc MOI = 0.01). To investigate the influence on viral attachment, pre-chilled Huh-7.5 cells were challenged with the HCVcc (MOI = 0.01) in the presence/absence of the test agent at 4 °C, which allows binding of the viral particles to the host cells while precluding entry. To test for impact on viral entry/fusion, Huh-7.5 cells were pre-bound with HCVcc (MOI = 0.01) at 4 °C and then shifted to 37 °C incubation in the presence/absence of the test drug. For all the above analyses, Huh-7.5 cells were seeded in 96-well plates (1 × 10^4^ cells/well) and luciferase activity for all conditions was determined as described earlier following 72 h of incubation at 37 °C.

### 2.6. Binding Assay

The enzyme-linked immunosorbent assay (ELISA)-based binding assay was performed as previously described [23]. Briefly, pre-chilled Huh-7.5 cells were challenged with HCVcc in the presence/absence of test drug at 4 °C for 3 h before washing with PBS and fixing the cells with 4 % paraformaldehyde. Cell-bound virus was detected using primary anti-HCV E2 antibody (1:20000; AUSTRAL Biological, San Ramon, CA, USA) and secondary goat anti-mouse IgG conjugated with horseradish peroxidase antibody (1:36000, Invitrogen), followed by assessment with TMB (3,3’,5,5’-tetramethylbenzidine) Two-component Microwell Peroxidase Substrate Kit (KPL; Gaithersburg, MD, USA) and absorbance reading at 450 nm with a ELx800 microplate reader (Instrument, Inc.; Winooski, VT, USA).

### 2.7. Analysis Using HCV Pseudoparticles

Retroviral pseudoparticles bearing HCV glycoproteins E1/E2 were produced following a previously described method [24] with some modifications. A pcDNA3.1 plasmid vector (Invitrogen) containing complete HCV core and E1/E2 glycoproteins of the HC-J6CH strain (genotype 2a; NC_009823) was co-transfected in conjunction with the pNL.Luc.Env^−^R^+^ construct (Env-defective retroviral backbone encoding firefly luciferase; kindly provided by Dr. Éric A. Cohen) into 293T cells using OMNIfect™ (transOMIC Technologies Inc.; Huntsville, AL, USA). Supernatant containing the viral pseudoparticles was harvested and filtered (0.45 μm) before being concentrated using 25% (*v*/*v*) polyethylene glycol and stored at −80 °C before use. For the infectivity experiment, the HCV pseudoparticles (HCVpp) were used to inoculate Huh-7.5 cell monolayers in 12-well plates in the presence or absence of the test agent for 2 h at 37 °C. The cells are then washed with PBS and further incubated in basal media for 72 h before being harvested and assessed for reporter luciferase activity using the Firefly Luciferase Assay kit (Promega) and a luminometer. Viral infectivity was calculated as percent (%) relative light units (RLU) compared to control (virus only).

### 2.8. Western Blot

Cells were lysed with RIPA buffer supplemented with protease inhibitors (Roche Molecular Biochemicals; Indianapolis, IN, USA) and subjected to standard immunoblotting using anti-CD81 (1:1000; BD Biosciences, San Jose, CA, USA), anti-CLDN-1 (1:1000; Invitrogen), anti-SR-BI (1:1000; Abcam, Cambridge, UK), anti-OCLN (1:200; Cell Signaling Technology, Danvers, MA, USA), anti-Apolipoprotein B (ApoB, 1:5000; Abcam), anti-ApoE (1:5000; Calbiochem-Millipore, Billerica, MA, USA), and anti-β-actin (1:20000; Santa Cruz Biotechnology, Dallas, Texas, USA) primary antibodies, and anti-mouse (1:5000; Invitrogen) or anti-rabbit (1:2500; Sigma) secondary antibodies. Imaging was performed on an UVP chemiluminescence imaging system (UVP; Upland, CA, USA).

### 2.9. Inhibitory Effects Against Multiple Genotypes of HCV

Antiviral activity against recombinant HCV carrying glycoproteins from genotype 2b (J8/JFH1), 3a (S52/JFH1), and 7a (QC69/JFH1) viruses (kindly provided by Dr. Jens Bukh) [25,26] was performed using a previously reported method [23] with some modifications. Huh-7.5 cells (1 × 10^4^ cells/well in 96-well plates) were challenged with the viruses at MOI = 0.01 in the presence/absence of the test agent. After 3 days of further incubation, detection of viral infectivity was performed by immunofluorescence staining of HCV foci using primary mouse anti-core clone B2 antibody (1:200; Anogen; Mississauga, ON, Canada), which cross-reacts different HCV core antigenic determinants, and a secondary Alexa Fluor 488 goat anti-mouse IgG (H+L) (1:400; Invitrogen) [27]. HCV-positive foci were quantitated and results were plotted against the DMSO control [28].

### 2.10. Statistical Analysis

Statistical analysis was conducted using one-way analysis of variance (ANOVA) followed by Tukey’s multiple comparison test or unpaired *t* test. A *p* value of less than 0.05 (*p* < 0.05) was considered to be statistically significant. All data are expressed as means ± standard error of the means (SEM) from three independent experiments.

## 3. Results

### 3.1. RC is Capable of Inhibiting HCV Infection

**Figure 1:**
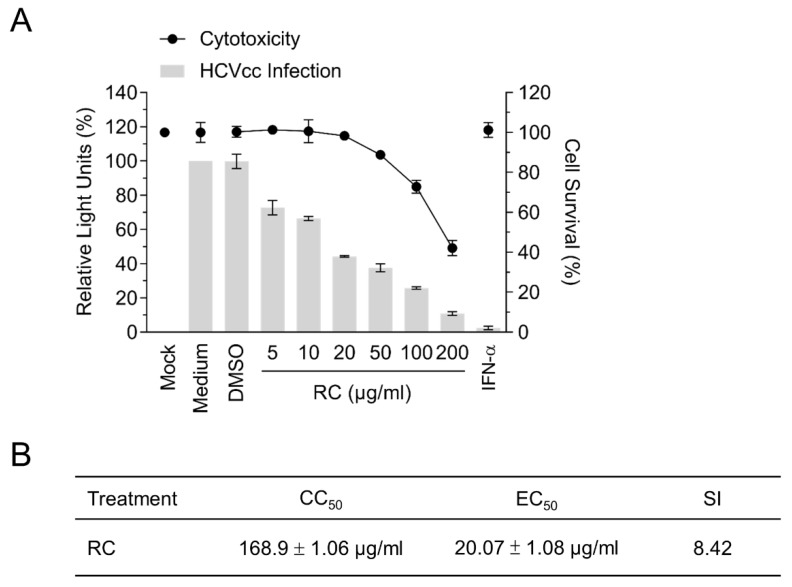
Analysis of RC’s antiviral activity against HCV infection in Huh-7.5 hepatoma cells. (**A**) Dose-response analysis of the cytotoxicity (relative to mock control) and antiviral efficacy (relative to medium control) of RC against HCV infection; IFN-α (800 IU/mL) served as positive control. (**B**) CC_50_, EC_50_, and SI values determined from A. Data represent means ± SEM from 3 independent experiments.

Given the numerous pharmacological properties of RC, we hypothesized that the medicinal herb could potentially possess antiviral activities against HCV. To explore this possibility, Huh-7.5 cells were infected with *Gaussia* luciferase reporter-tagged HCVcc in the presence of different concentrations of the methanolic extract of RC and the luciferase activity was subsequently assessed to determine viral infectivity. A cytotoxicity assay was simultaneously performed on the cells with the same drug concentrations. Our results showed that RC could inhibit HCV infection dose-dependently and up to 50 μg/mL without inducing significant cytotoxicity (Figure 1A). The 50 % cytotoxic concentration (CC_50_), the 50 % effective concentration (EC_50_), and the selective index (SI = CC_50_/EC_50_) were determined to be 168.9 ± 1.06 μg/mL, 20.07 ± 1.08 μg/mL, and 8.42, respectively (Figure 1B). Since the antiviral efficacy of RC appeared to be comparable between 20 and 50 μg/mL, a concentration of 20 μg/mL was chosen for the remainder of our experiments.

### 3.2. RC Inhibits the Early Steps of HCV Entry

**Figure 2:**
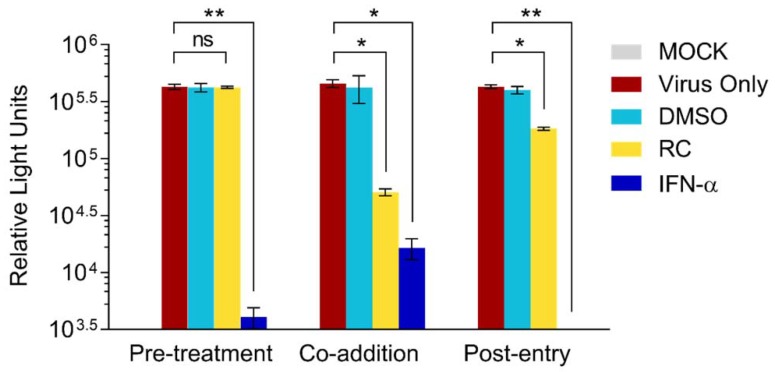
Time-of-drug-addition analysis of RC’s antiviral effect. All data represent means ± SEM from 3 independent experiments. RC = 20 μg/mL; DMSO = 0.5%; IFN-α = 800 IU/mL; *p < 0.05, **p < 0.01, ns: not significant.

In order to narrow down the window of antiviral activity from RC, we performed a time-of-drug-addition assay wherein the drug was either added to cells 24 h before HCVcc infection (“pre-treatment”), concurrently added at the time of viral infection (“co-addition”), or added after infection (“post-infection”) and incubated for 3 days before measuring the luciferase reporter activity. Results indicated no significant difference in HCV infectivity in the presence or absence of RC during pretreatment (Figure 2), suggesting that the drug does not appear to modulate the host cells before HCV infection. In contrast, RC significantly inhibited HCV infectivity when simultaneously added during the viral challenge as indicated by the substantial drop in the luciferase signal (Figure 2). Only a moderate decrease in viral infectivity was observed when the drug was added after the establishment of viral infection. In contrast, IFN-α, which served as positive control, effectively inhibited the HCVcc infection in all 3 types of treatment. Thus, RC’s anti-HCV activity appeared strongest when concurrently present on the host cell with the viral particles, suggesting that its inhibitory effect mainly targeted the early phase of the HCV infection, including viral entry.

### 3.3. RC Blocks HCV Viral Attachment and Entry/Fusion into the Host Cells

**Figure 3:**
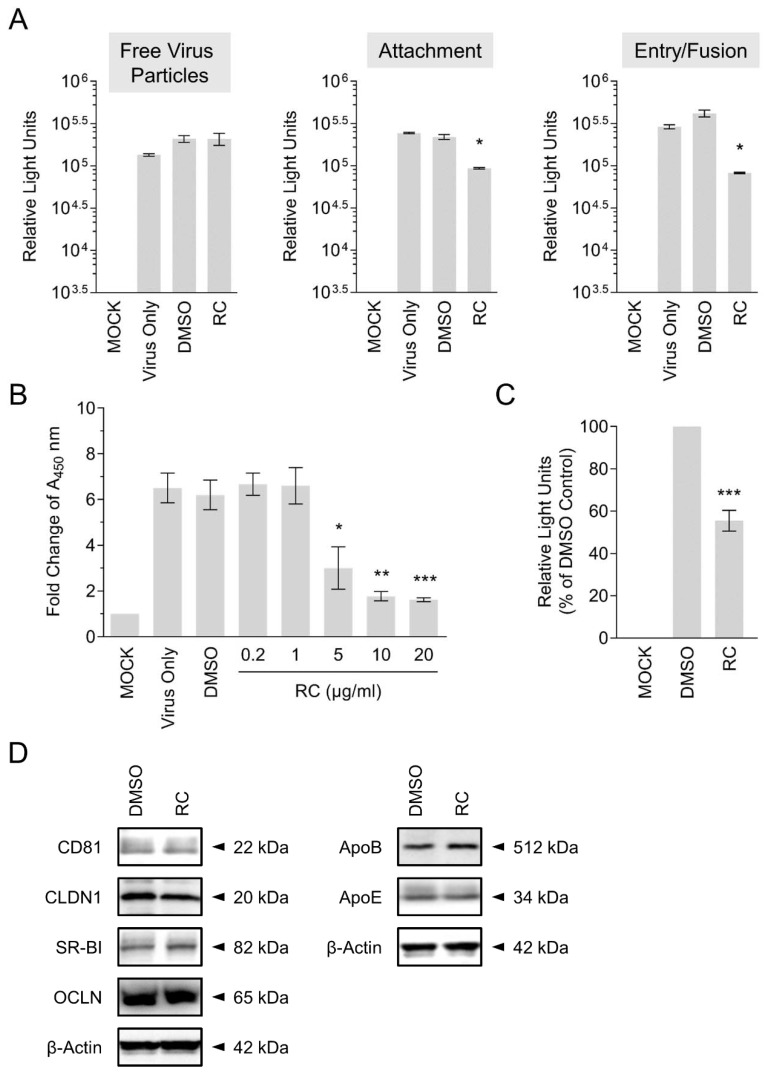
Investigation of RC’s antiviral effect on HCV entry. (**A**) Synchronized infection analysis of RC treatment on HCV early viral entry. (**B**) Validation of RC’s inhibitory activity against HCV attachment using ELISA-based virus binding assay. (**C**) Impact of RC treatment on HCVpp infection of Huh-7.5 cells. (**D**) Western blot analysis of RC treatment effect on HCV host cell entry factors. Data represent means ± SEM from 3 independent experiments. RC = 20 μg/mL unless otherwise indicated; DMSO = 0.5 %; *p < 0.05, **p < 0.01, ***p < 0.001. For Western blot analysis, representative blots from 3 independent experiments are shown. β-actin served as loading control.

To further characterize the mechanism(s) underlying RC’s anti-HCV effect, which was strongest when RC was simultaneously present with the virus on the host cell surface, we performed a synchronized infection assay on early viral entry. To test whether RC could inactivate the cell-free viral particles, the drug was pre-incubated with the HCVcc for 3 h before dilution and infecting Huh-7.5 cells. The drug-virus mixture was then diluted 20X to yield a non-effective concentration which was subsequently added to the cells and incubated for 3 days. Luciferase readings following 3 days of incubation showed no significant difference between samples treated with or without RC, suggesting that the drug does not impact the free viral particles (Figure 3A). To determine the effect of RC on viral attachment, we specifically added RC during HCV cell binding at 4 °C, which allows for virus binding but precludes viral internalization, and then tested the reporter readout at the end of the incubation. Results demonstrated a significant decrease in the luciferase reporter activity, which indicated that RC interfered with the attachment of the virus to the host cells (Figure 3A). To ascertain whether RC influenced the post-attachment viral entry/fusion step, Huh-7.5 cells were pre-bound with HCV at 4 °C before shifting the temperature to 37 °C in the presence of RC. Similar to the effect observed on viral attachment, our data revealed a significant decrease in HCV infectivity at the end of the incubation, suggesting that RC equally blocked HCV entry/fusion (Figure 3A).

### 3.4. Confirmation of RC’s Antagonism Against HCV Cell Attachment by ELISA-Based Virus Binding Assay

To directly validate our finding that RC inhibits HCV attachment to the host cells, we employed an ELISA-based binding assay. Huh-7.5 cells were inoculated with HCVcc in the presence of RC at 4 °C for 3 h, after which the cells were washed, fixed, and subjected to colorimetric analysis by detecting cell surface-bound HCV particles using anti-HCV E2 antibody. As depicted in Figure 3B, RC treatment dose-dependently decreased viral attachment as demonstrated by the sharp decline in the absorbance signal, which is in agreement with the above results.

### 3.5. RC Robustly Inhibits Infection by Pseudoparticles Bearing HCV Glycoproteins

HCV entry steps are mediated by viral glycoproteins [2]. Based on the inhibitory effects observed from RC against HCV entry steps, and to validate its antiviral potency against HCV entry, we further examined the impact of RC treatment on infection by retroviral pseudoparticles bearing HCV glycoproteins E1/E2 (HCVpp). Specifically, luciferase-tagged HCVpp were used to infect Huh-7.5 cells in the presence or absence of RC, before further incubating the cells and assessing luciferase reporter activity as a readout of viral infectivity. As demonstrated in Figure 3C, RC robustly suppressed HCVpp infection of the Huh-7.5 hepatoma cells compared to the DMSO control. This result therefore confirms and validates the antiviral capacity of RC against the viral entry stage of HCV.

### 3.6. RC Does Not Modulate Host Cell Entry Factors to HCV Infection

HCV infection of hepatocytes is a well-orchestrated process involving the engagement of several host factors including CD81, SR-BI, CLDN-1, and OCLN. In addition, ApoE has also been reported to play a role in mediating HCV entry [5]. Given that RC treatment blocked HCV infection mainly by targeting viral entry, we next asked whether the drug could modulate the expression of key host cells entry factors. To this end, Huh-7.5 cells were pre-treated with RC for 24 h before harvesting the cell lysates for Western blot analysis. As depicted in Figure 3D, pre-treatment of Huh-7.5 cells with RC did not alter the expression of CD81, SR-BI, CLDN-1, and OCLN. Similarly, the expression of ApoE was not affected by RC treatment (Figure 3D). ApoB, which participates in HCV virion release was included for comparison and was neither affected by the drug treatment. These results therefore suggested that RC did not inhibit HCV infection via influencing the host cells and is consistent with our data from the pre-treatment analysis (Figure 2).

### 3.7. RC Inhibits Multiple HCV Genotypes

**Figure 4:**
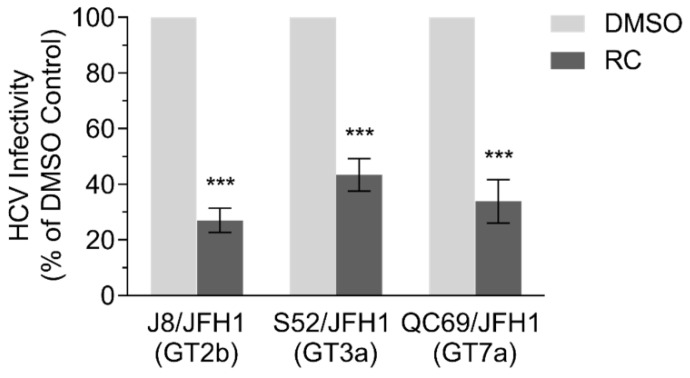
Inhibitory effect of RC treatment on multiple HCV genotypes. All data represent means ± SEM from 3 independent experiments. RC = 20 μg/mL; DMSO = 0.5 %; ***p < 0.001, unpaired *t* test.

Since RC appeared to primarily target HCV entry, we finally sought to examine whether the medicinal herb also possesses a pan-genotypic activity against HCV. For this purpose, Huh-7.5 cells were seeded and infected with recombinant HCVcc expressing glycoproteins from genotypes 2b (J8/JFH1), 3a (S52/JFH1), and 7a (QC69/JFH1) in the presence of the RC before further incubation and analysis of HCV infectivity by immunofluorescence staining. Results showed a significant decrease in viral infection across all the tested HCV genotypes when RC was present, suggesting that it may possess a pan-genotypic activity against HCV (Figure 4).

## 4. Discussion and Conclusions

Continuous identification of novel antivirals with various modes of action is important given that development of drug resistance is commonplace especially with viruses that exhibit genetic variability, including HCV. In this study, we demonstrated for the first time that the methanolic extract of RC robustly inhibited HCV infection. Specifically, RC mainly targeted the HCV early viral entry steps such as attachment and entry/fusion to the host cells. Interestingly, RC also inhibited HCV infection of several other genotypes. Our results therefore identified RC as a promising antagonist with pan-genotypic function against HCV entry, which could be useful for developing HCV prophylaxis.

Currently no approved therapeutic treatment exists for targeting HCV entry, and patients with chronic hepatitis C are at risk for end-stage liver diseases such as cirrhosis and liver cancer, which necessitate liver transplantation. Importantly, donor livers inadvertently become re-infected almost immediately after transplantation in hepatitis C patients [29]. Given that the DAAs in current use cannot prevent liver graft re-infection and have the propensity to select for drug-resistant mutants, combining entry inhibitors with the DAAs would be expected to broaden the treatment strategies against hepatitis C especially in the liver transplant setting. Interestingly, previous studies have demonstrated that combining DAAs and virus entry inhibitors can produce a synergistic effect to improve drug efficacy [30]. Our discovery that RC can antagonize HCV entry makes it an ideal candidate for use in liver transplant scenarios and to test in combination with the DAAs for better therapeutic efficacy.

Natural resources such as plant materials serve as excellent starting point for antiviral discovery [31]. RC’s anti-HCV bioactivity identifies the medicinal herb as an important antiviral source for the treatment of hepatitis C. Various medicinal plant extracts have been shown to possess anti-HCV properties and thereafter served as source for further identifying small molecule inhibitors. Examples of those that specifically target HCV entry include silibinin and silymarin from *Silybum marianum* [32,33], gallic acid found in *Limonium sinense* [22], loliolide and the butenolide (4R,6S)-2-dihydromenisdaurilide derived from *Phyllanthus urinaria* [28,34], ladanein isolated from *Marrubium peregrinum* L. (Lamiaceae) [35], and *Bupleurum kaoi* and its terpenoid saikosaponin b2 [23]. While the molecular constituents in RC contributing to its anti-HCV activity remain to be identified, the alkaloids are potential candidate bioactives which are known to be the major components of RC and are typically present in the alcoholic extracts of the herb [11]. Examples include berberine, coptisine, palmatine, epiberberine, columbamine, jatrorrhizine, and groenlandicine, among other major compounds [11]. Whether a single compound or a combination of them contributes to RC’s antiviral actions against HCV remains to be explored. Further in-depth analysis would be required to fully elucidate the bioactive ingredients responsible for RC’s anti-HCV effect.

Our results indicated that RC specifically blocked HCV attachment and entry/fusion into the host cells without significantly influencing the cell-free viral particles or modulating key host cell entry factors to HCV. The ability of RC to inhibit HCVpp infection suggests that its mechanistic target(s) may involve the HCV glycoproteins-mediated interactions with the host cell. A potential mechanism is the transient interaction(s) with the HCV glycoproteins that are inadequate to inactivate free viral particles but sufficiently effective to hinder contact with cell surface receptors/co-receptors. Additional mechanisms of action could also include concentration-dependent reversible conformational alterations in receptors/co-receptors or viral particles’ structure to block viral entry. We also noted a moderate effect from RC in the post-infection stage (Figure 2), albeit less pronounced compared to its impact in inhibiting HCV entry steps. This effect was not entirely due to inhibition of entry in subsequent de novo infection cycles, since treatment of subgenomic HCV replicon cells with RC also showed a moderate decrease in viral RNA in a preliminary experiment (Figure S1). It is possible that RC may possess multiple mechanisms against HCV infection, which would necessitate further studies for clarification. Nonetheless, our results clearly demonstrated that RC most potently inhibited HCV entry. Therefore, we suggest that RC could be further explored for prophylactic/therapeutic management of hepatitis C.

## Supplementary Materials

The following are available online at http://www.mdpi.com/1999-4915/10/12/669/s1, Figure S1: Effect of RC treatment on HCV subgenomic replicon cells.
