# Supplementary material for: Methanolic Extract of Rhizoma Coptidis Inhibits the Early Viral Entry Steps of Hepatitis C Virus Infection
Source: Viruses. 2018 Nov 27;10(12):669. doi: 10.3390/v10120669 (PMC6315547; doi:10.3390/v10120669)
Supplement: Supplementary file 1 [file viruses-10-00669-s001.pdf]

Supplementary Material

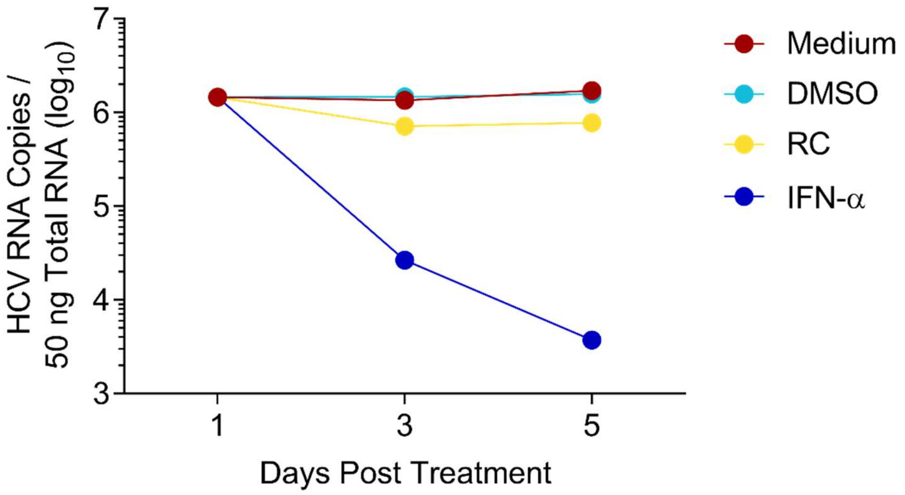

Figure S1. Effect of RC treatment on HCV subgenomic replicon cells. The sbJFH1-B2 subgenomic replicon cells (JFH1; genotype 2a) [23] were treated with or without RC (20 μg/mL) for 1, 3, and 5 days before harvesting the cells and extracting total RNA using TRIzol (Invitrogen). Quantitation of HCV genome by qRT-PCR (input of 50 ng of total cellular RNA per sample) was performed as previously described [23]. DMSO = 0.5%; IFN-α (800 IU/mL) served as positive control.
